# Extract of the Notes Subjoined to the Translation of Dr. Reid's Essay, on the Nature and Treatment of Pulmonary Consumption

**Published:** 1799-08

**Authors:** F. L. Dumas

**Affiliations:** Professor of the School of Medicine, at Montpelier


### *Extract of the Notes subjoined to the Translation of* Dr. Reid's *Essay, on the nature and treatment of Pulmonary Consumption:—By* F. L. Dumas, *Professor of the School of Medicine, at Montpelier.*

Those subjects who have lungs liable to an excessive irritability, whether natural or acquired, labour under a sort of phthisis, the particular character of which is a spasmodic irritation in the pulmonary organs, or in the membranes which surround them; whence a quick and habitual contraction results, which strains and dries the parts, so as nealy to tear the parenchyma, or which at least hardens them sometimes into scirrhosities, or tubercles, the formation of which is almost generally a prelude to the total decomposition of their substance. This kind of phthisis, peculiar to persons who have long been subject to nervous and convulsive coughs, who experience frequent attacks of the dry asthma, and are habitually exposed to respire metallic, irritable, and corrosive vapours, is indicated by a dry and sonorous cough, which is not followed by a free expectoration. The texture of the skin is closed, gross, and rather contracted, the voice harsh, the respiration constrained, the sensibility increased, the pulse hard, and like the vibrations of a stretched cord; nervous symptoms of vapours are apparent, which promote the continuance of the general symptoms, common in all consumptions. Examples of that species dependent on nervous irritation, are to be found in the works of Morton, Willis, Vogel and Baglivi, &c. Morton, vol. 1. book 3. chap. iii. Willis, Pharmac. Rat. Sec. 1. chap. 6.

Pure or dephlogisticated air, or oxygen gas, recommended by some physicians in the treatment of certain kinds of consumption, would be absolutely deleterious, and productive of dissolution, in the species under consideration. I believe I may assert, upon the authority of experience, that the continual use of such air would introduce into the lungs that acute degree of irritation, which which often gives rise to tubercles, or to the ulcerous state of which this disease is the result. I have, with some attention, continued the experiments made with this view on animals, and I am convinced that, when submitted for some length of time to the impression of oxygen gas, the lungs become irritated, inflamed, grow red, and suppurate, which is the cause of consumption. I shall now give an account of some experiments I have made on living animals, which no other person ever before attempted.

I took a dog, of middle size, in a perfect state of health, and placed him under a large receiver, from which the atmospheric air was expelled, and which was filled with oxygen gas. I adapted two tubular syphons to the receiver, and had a cock fitted to the tube, which I could open and shut at pleasure. I used one of the syphons to discharge the air impregnated with oxygen gas, as it became foul by the respiration of the animal, the other opened into the receiver, to renew, when requisite, the quantity of oxygen gas which had evaporated, so that by means of my two syphons, I was enabled to keep the air in the receiver pure, and to preserve in it at all times the same quantity of oxygen. My apparatus thus prepared, and the dog put under the receiver, previously filled with oxygen, I let him remain in this atmosphere, which he respired without any addition of air, during the space of six hours. At the expiration of this time, the respiration appeared to be accelerated, and the animal began to show signs of uneasiness. I then withdrew him, and placed him in an atmosphere better adapted to his nature. In the evening I repeated the same experiment, which I did constantly twice a day till the 28th, when his lungs ceased to move with their usual facility. I found it necessary to shorten the time of the experiment, and I could not continue it fifteen days longer without the greatest difficulty. At this time the animal had almost entirely lost the power of breathing and howling; his respiration became sonorous, weak, and painful; the sounds of his voice were hoarse and stifled; his eyes appeared dim and languishing; he suddenly shed a great quantity of hair, particularly from about the breast; he lost his flesh very rapidly; and perceiving in him all the symptoms of an approaching consumption, I resolved to kill him, and open the thorax, to examine the state of his lungs, and ascertain what I had before suspected. The cavity of the breast being laid open, I found its right side filled with an acrid serosity, containing much coagulated blood. The serous humour, when thrown upon live coals, evaporated in the air, except a pellicle which appeared in the form of a bladder. The coagulated blood appeared of a fleshy consistence, similar to that of the pleuritic sarcoma, and was situated near the superior part of the lungs, corresponding with the bronchiæ. The bronchial vessels also appeared distended; the pleura likewise lightly adhered to to the lungs, particularly in their inferior part, which was at the same time caked in all the adjacent parts; this membrane was red, swelled and inflamed. The lungs, which were reddish and intersected with little tears, had acquired a considerable hardness, as happens to those organs which have remained a long time inflamed. I perceived in the bronchiæ a small wound, the hard and callous edges of which indicated it would soon have degenerated into an ulcer. The anatomical inspection of those parts, no longer let me doubt that the oxygen had given an irritable impression to the lungs, from whence had resulted all the ordinary symptoms of consumption.

I wished to try the same experiment again, and had fixed upon the time when I was obliged to attend to other occupations, which prevented me from effecting it; but it was my intention to resume it, with variations, as soon as I should have the least leisure. This consumption, depending on an acute irritation of the pulmonary organs, would doubtless be accelerated by an active and more powerful method of exhibiting the gas. Riding, which Sydenham has so much recommended, the active resolvents, the sulphur, brought into notice by Dr. Sims, myrrh, bitter plants, bathing, and kino, are so many poisons, which, in cases of this nature accelerate death, that might be prevented by proper remedies. It is needless to observe, that the use of emetics, or small doses of ipecacuanha, and consequently the method of Dr. Reid, bring on the same dangers, and ought to excite in us similar apprehensions.

If the lungs are afflicted with weakness, or such a deep atony as to be unable to perform their functions, suffocation is to be apprehended, and a collection of matter which forms tubercles, and produces another species of consumption, of which the essential cause will be a radical nervous weakness of the lungs. This languishing organ, becomes at length incapable of acting; the absorption and exhalation which ought to be made by it, are diminished; the blood does not receive the necessary quantity of oxygen, that vivifying principle, that *pabulum vitæ*, which seems destined to unite the principles of the blood, and to repair the elements of the muscular fibres; the sanguification remains imperfect, vitiated, and only exhibits a pale and weak blood, which, not participating in the digestive assimilation, cannot furnish the body with a sufficient degree of nourishment; the limbs grow dry and fall into consumption; the lungs remain inert, and are unable to eject the matter exhaled; a certain quantity of this matter, retained by the defect of a proportionate secretion, accumulates in the lungs, from whence it returns to the general passages of circulation, and occasions a hectic, pulmonary fever. This species of consumption is manifest from a dry and lean state of the body, the dilatation of the cellular membranes, a paleness, ness, debility, hoarseness of voice, softness of the skin, frequent and difficult expectoration, an œdematous state of the extremities, lassitude, languor, enervation of the whole body, &c.

Hereditary consumptions commonly have symptoms similar to the latter species, and nothing contributes to produce them so much as respiring for a long time in a thick, gloomy air, loaded with miasmata, or vitiated by the combination of some deleterious gas. I have begun a series of experiments on this subject, from which I can at present only produce those which confirm the effect of the carbonic acid gas on the lungs, which leave no doubt of the existence and nature of the consumption which it occasions.

I placed two dogs, under two receivers, filled with carbonic acid gas, and contrived in such a manner that I could introduce, when necessary, a certain quantity of pure air, so as to make it the longer respirable, and likewise to prolong the pernicious impression on the organs of the two animals. They remained under these receivers till they began to shew signs of uneasiness, and I then only withdrew them that I might again submit them, at a future time, to the effects of the deleterious gas. I alternately placed and withdrew them several times in the same day, and repeated the same experiments alternately every day, during the space of six weeks. These animals became by degrees incapable of respiring such an atmosphere. One of them, whose lungs would not receive the deleterious gas, died under an experiment. The other I killed, as soon as he manifested signs of weakness. I observed, before their death, that they both experienced a difficulty of respiration, a weak and hoarse voice, foamed at the mouth, and their whole body appeared considerably emaciated. I opened the body of the first, and examined particularly the organs of the chest. I found the lungs adhering to the pleura of the left side; the other parts of the lungs which remained free were filled with a lymphatic and serous matter, in which floated several shreds of gelatinous, or rather membranous substances. The lungs were covered with black spots; they were of a pulpous texture; their size had decreased so that they were scarcely equal to those of an animal just born. I perceived clots of blood deposited at the orifice of the pulmonary vessels: the other cavities were perfectly sound.

The lungs of the second animal only exhibited a multiplicity of adherences, and I clearly perceived that I had let him perish before the gas had sufficiently operated. I intend to repeat my experiments with azotic gas.

The method of Dr. Reid, which is capable of giving a tonic impression to the lungs, would, in this species of consumption, possess all the advantages which are derived from other renovating, and strengthening remedies.

